# The use of PCR/Electrospray Ionization-Time-of-Flight-Mass Spectrometry (PCR/ESI-TOF-MS) to detect bacterial and fungal colonization in healthy military service members

**DOI:** 10.1186/s12879-016-1651-7

**Published:** 2016-07-22

**Authors:** Ryan Vetor, Clinton K. Murray, Katrin Mende, Rachel Melton-Kreft, Kevin S. Akers, Joseph Wenke, Tracy Spirk, Charles Guymon, Wendy Zera, Miriam L. Beckius, Elizabeth R. Schnaubelt, Garth Ehrlich, Todd J. Vento

**Affiliations:** San Antonio Military Medical Center, JBSA Fort Sam Houston, San Antonio, TX USA; Uniformed Services University of the Health Sciences, Bethesda, Maryland USA; Infectious Disease Clinical Research Program, Uniformed Services University of the Health Sciences, Bethesda, MD USA; Center for Genomic Sciences, Allegheny Singer Research Institute, Pittsburgh, PA USA; US Army Institute of Surgical Research, Fort Sam Houston, San Antonio, TX USA; Landstuhl Regional Medical Center, Landstuhl, Germany; Infectious Disease Service, (MCHE-MDI), Brooke Army Medical Center, 3551 Roger Brooke Drive, JBSA Fort Sam Houston, 78234 Texas, USA

**Keywords:** Colonization, Bacterial, Fungal, Molecular diagnostics, Military, PCR, Electrospray ionization time-of-flight mass spectrometry

## Abstract

**Background:**

The role of microbial colonization in disease is complex. Novel molecular tools to detect colonization offer theoretical improvements over traditional methods. We evaluated PCR/Electrospray Ionization-Time-of-Flight-Mass Spectrometry (PCR/ESI-TOF-MS) as a screening tool to study colonization of healthy military service members.

**Methods:**

We assessed 101 healthy Soldiers using PCR/ESI-TOF-MS on nares, oropharynx, and groin specimens for the presence of gram-positive and gram-negative bacteria (GNB), fungi, and antibiotic resistance genes. A second set of swabs was processed by traditional culture, followed by identification using the BD Phoenix automated system; comparison between PCR/ESI-TOF-MS and culture was carried out only for GNB.

**Results:**

Using PCR/ESI-TOF-MS, at least one colonizing organism was found on each individual: mean (SD) number of organisms per subject of 11.8(2.8). The mean number of organisms in the nares, groin and oropharynx was 3.8(1.3), 3.8(1.4) and 4.2(2), respectively. The most commonly detected organisms were aerobic gram-positive bacteria: primarily coagulase-negative *Staphylococcus* (101 subjects: 341 organisms), *Streptococcus pneumoniae* (54 subjects: 57 organisms), *Staphylococcus aureus* (58 subjects: 80 organisms) and *Nocardia asteroides* (45 subjects: 50 organisms). The *mecA* gene was found in 96 subjects. The most commonly found GNB was *Haemophilus influenzae* (20 subjects: 21 organisms) and the most common anaerobe was *Propionibacterium acnes* (59 subjects). *Saccharomyces* species (30 subjects) were the most common fungi detected*.* Only one GNB (nares *E. coli*) was identified in the same subject by both diagnostic systems.

**Conclusion:**

PCR/ESI-TOF-MS detected common colonizing organisms and identified more typically-virulent bacteria in asymptomatic, healthy adults*.* PCR/ESI-TOF-MS appears to be a useful method for detecting bacterial and fungal organisms, but further clinical correlation and validation studies are needed.

## Background

Our understanding of the nature of host-microbe interactions has become increasingly complex, with the line between pathogen and non-pathogen becoming more and more obscure [1]. Individual organism and human factors, in addition to complex interactions between host and pathogen, play significant roles in determining the colonization-infection-disease spectrum [1–6]. Historically, the role of the human microbiome in disease has been poorly understood, partly due to limitations of standard microbiological culture [6–8]. Recently, novel molecular platforms such as PCR/Electrospray Ionization-Time-of-Flight-Mass Spectrometry (PCR/ESI-TOF-MS) have shown potential benefits over standard microbiological culture, including increased throughput and cost savings [9]. PCR/ESI-TOF-MS offers not only microbiologic organism identification, but the capability to detect genetic resistance elements. This molecular tool has also shown high concordance with traditional culture methods in identifying microorganisms in blood cultures [10] and in different orthopedic populations [11, 12]. There have been only a few studies using PCR/ESI-TOF-MS to explore colonization rates, with some published surveillance studies from a burn intensive care unit and orthopedic ward healthcare workers, and preliminary data on staphylococcal colonization in United States military personnel [13, 14]. Given the potential link between colonization and modification of the microbiome to subsequent infections, this pilot study sought to examine bacterial and fungal colonization of healthy individuals using PCR/ESI-TOF-MS, to further understand human microbiome diversity using a newer molecular platform, and to compare gram-negative bacteria (GNB) detection by PCR/ESI-TOF-MS with traditional culture.

**Fig. 1:**
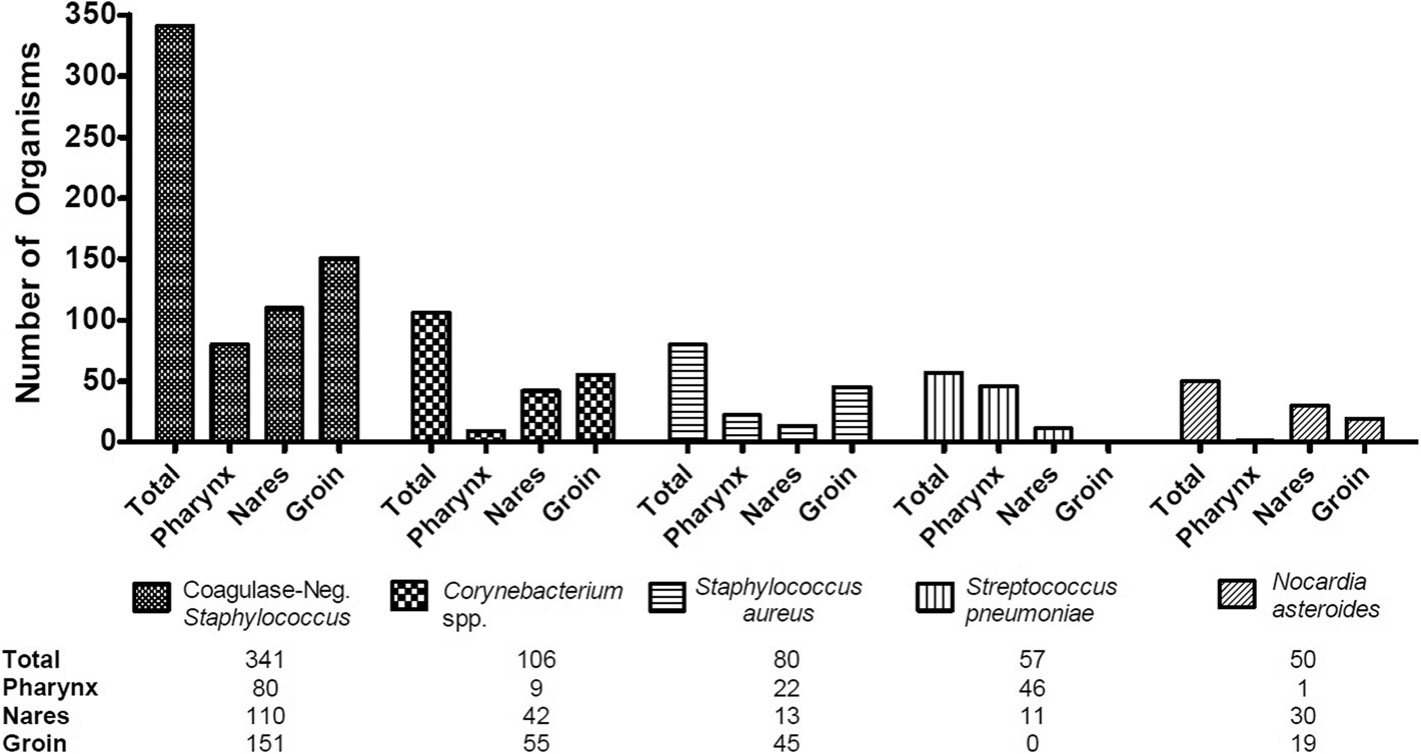
Five most commonly detected gram-positive bacteria (from healthy service members) by PCR/ESI-TOF-MS

**Fig. 2:**
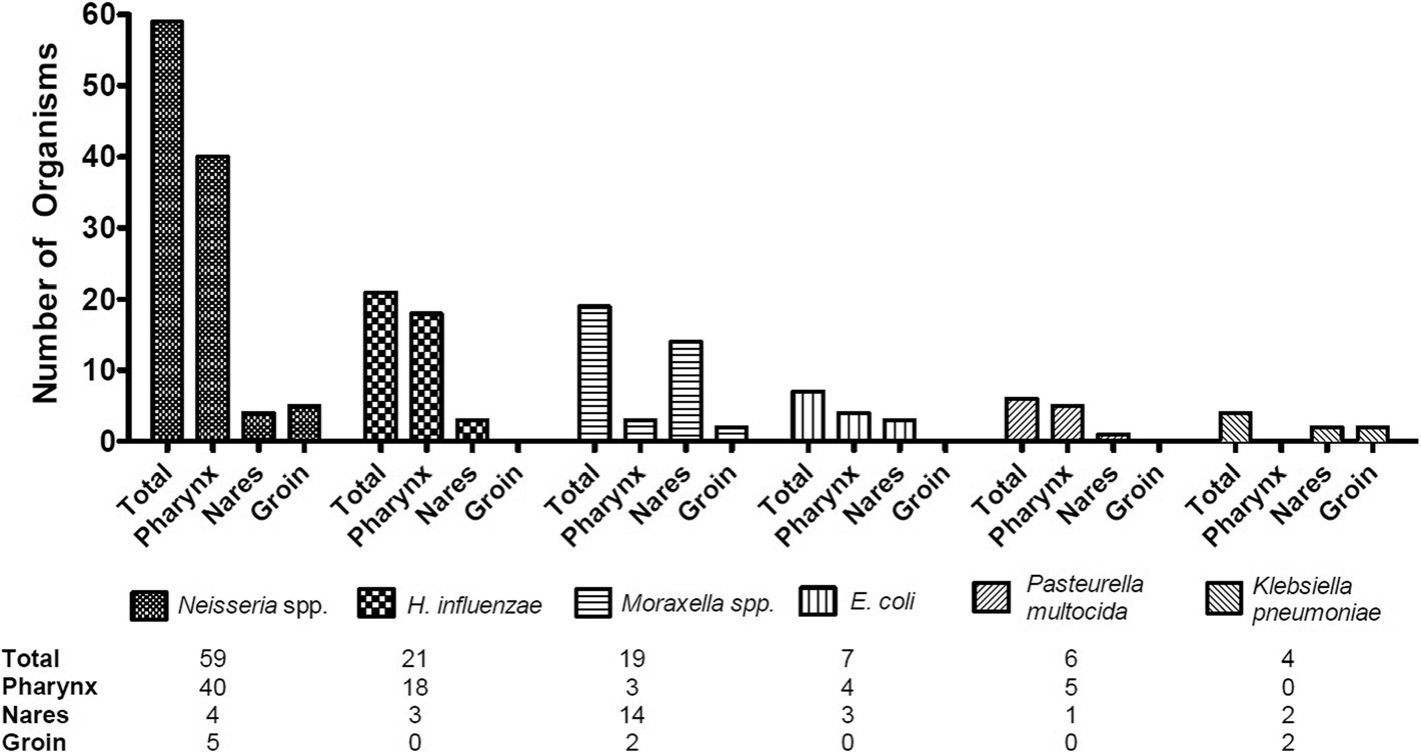
Six most commonly isolated gram-negative bacteria (from healthy service members) by PCR/ESI-TOF-MS

## Methods

### Study population

One hundred one healthy active duty service members presenting to the outpatient Troop Medical Clinic for acute care at Fort Sam Houston, TX were enrolled. Study participants were 18 years and older who had no overseas travel or deployment within the past 6 months, no antibiotic use in the preceding 30 days, and were being seen for non-infectious medical conditions. Written informed consent was obtained from study participants upon enrollment. Individuals were excluded from the study if they were deemed to have an acute or chronic infection that may have altered normal bacterial flora or that involved a proposed anatomic sample site. The study protocol was approved by the San Antonio Military Medical Center (SAMMC) Institutional Review Board. The results presented here are a sub-study of a larger protocol examining various methods for detecting microbial colonization in healthy US military personnel; initial results on methicillin-resistant and methicillin-sensitive *Staphylococcus aureus* (MRSA and MSSA) and multidrug-resistant (MDR) GNB colonization have been published [14, 15]. Our current study further examines bacterial and fungal colonization of healthy U.S. military individuals, including the correlation of GNB detection by PCR/ESI-TOF-MS and traditional culture.

### Specimen collection and processing

Individuals were screened with culture swabs (Copan Stuart liquid media culture, Copan Inc., Brescia, Italy) from various anatomic sites (nares, oropharynx, and groin). Each participant was sampled by application of the swab in a repetitive twisting motion at each anatomic site, firmly brushing the skin or mucosa to ensure transfer of cells onto the swab tip. Samples for PCR/ESI-TOF-MS were frozen at -20^o^ C or lower and transported on dry ice for batched testing to the Center for Genomic Sciences, Allegheny Singer Research Institute, Pittsburgh, PA, USA. A separate set of swabs collected in the same fashion were transported to the SAMMC research microbiology laboratory and underwent immediate processing for microbial culture to identify GNB. The analysis of coagulase-negative *Staphylococcus* (CNS) and *S. aureus* detection by traditional culture and PCR/ESI-TOF-MS methods has been published elsewhere [14]. We did not evaluate other gram-positive bacteria, fungus or anaerobes by traditional culture due to cost constraints.

### PCR/ESI-TOF-MS

Frozen swabs were thawed after transport, placed in microcentrifuge tubes containing 270 μl of ATL Lysis buffer (Qiagen, Germantown, MD, cat# 19076) and 30 μl proteinase K (Qiagen, cat# 19131) and then incubated at 56 °C for 1 h. Next, 100 μl of a mixture containing 50 μl each of 0.1 mm and 0.7 mm Zirconia beads (Biospec, Bartlesville, OKcat# 11079101z, 11079107zx, respectively) were added to the samples which were then homogenized for 10 min at 25 Hz using a Qiagen Tissuelyser. Nucleic acid from the lysed sample was then extracted using the Qiagen DNeasy kit (Qiagen cat# 69506). 10 μl of each sample was loaded per well onto the BAC detection PCR plate (Abbott Molecular, Carlsbad, CA cat# PN 05 N13-01). The BAC detection plate contains 96 wells each with 16 primers that survey all bacterial organisms by using multiple omnipresent loci (e.g., 16S rDNA sequences) and multiple pluripresent loci (e.g., the tufB gene). The BAC assay has been validated against 613 organisms [9]^.^ The system also detects the presence of several key antibiotic resistance markers: *vanA* and *vanB* (vancomycin resistance) in *Enterococcus* spp., KPC-3 (carbapenem resistance) in GNB, and *mecA* (methicillin resistance) in *Staphylococcus* spp. An internal calibrant of synthetic nucleic acid template is also included in each assay, controlling for false negatives (e.g., from PCR inhibitors) and enabling a semi-quantitative analysis of the amount of template DNA present. PCR amplification was performed according to Ecker et al. [9] The PCR products were then desalted in a 96-well plate format and sequentially electrosprayed into a time-of-flight mass spectrometer. The spectral signals were processed to determine the masses of each of the PCR products present with sufficient accuracy to determine the base composition of each amplicon. Using combined base compositions from multiple PCRs, the identities of the pathogens and a semi-quantitative determination of their relative concentrations in the starting sample were established by using a proprietary algorithm which interfaces with the Ibis database of known organisms.

### Traditional culture

All isolates were plated onto both Trypticase^™^ Soy Agar with 5 % sheep blood (sheep blood agar, BBL, Cockeysville, MD, USA) and MacConkey agar (BBL, Cockeysville, MD, USA) in order to isolate aerobic GNB colonies. Colonies grown on MacConkey agar and colonies consistent with gram-negative morphology on sheep blood agar were sub-cultured onto sheep blood agar to assure culture purity and then frozen at -80^o^ C for subsequent evaluation. Frozen isolates underwent two passages on sheep blood agar, followed by automated testing for species-identification and susceptibility via the BD Phoenix Automated Microbiology System (Becton Dickinson and Company, Franklin Lakes, NJ) using NMIC/ID-123 panels according to manufacturer’s guidelines.

### Analysis

Organisms were categorized based on taxonomy and morphological features. Basic descriptive statistics were used to summarize the findings using SPSS (IBM®SPSS®Statistics Version 19). Comparison between PCR/ESI-TOF-MS and traditional culture results was only performed on GNB utilizing Spearman’s rank correlation.

## Results

### Demographics

Three anatomic sites (nares, oropharynx, and groin) of 101 participants were swabbed, with one subject declining the oropharynx swab. The median age of participants was 23 [IQR 22, 23] years, with 69 % being male.

### PCR/ESI-TOF-MS

PCR/ESI-TOF-MS identified at least one organism in every subject, with a mean number of 11.8 [SD 2.8] organisms per subject. The most commonly deteceted gram positive and gram negative bacteria, by anatomic site, are shown in Figs. 1 and 2. The oropharynx site yielded the highest mean number of organisms, 4.2 [SD 2.0] followed by groin 3.8 [SD 1.4] and nares 3.8 [SD 1.3] (Table 1).

**Table 1:** Bacterial and fungal colonization of the nares, oropharynx and groin of 101 healthy service members detected by PCR/ESI-TOF-MS

	Total	Bacteria			Fungi
		Aerobic gram-positive	Aerobic gram-negative	Anaerobic	
Total colonized					
Subjects	101	101	80	75	46
Organisms	11.8 (2.8)	8.6 (2.0)	1.4 (1.0)	1.2 (1.0)	0.6 (0.8)
Nares colonization					
Subjects	101	99	31	56	13
Organisms	3.8 (1.3)	2.7 (1.3)	0.3 (0.5)	0.6 (0.6)	0.1 (0.4)
Oropharynx colonization					
Subjects	99	95	65	22	37
Organisms	4.2 (2.0)	2.6 (1.3)	0.9 (0.9)	0.3 (0.6)	0.4 (0.5)
Groin colonization					
Subjects	101	101	13	30	8
Organisms	3.8 (1.4)	3.3 (1.7)	0.1 (0.4)	0.3 (0.5)	0.1 (0.3)

Organisms presented as mean number of organisms per subject (standard deviation)

Overall, aerobic gram-positive bacteria were more prevalent than GNB (870 vs. 140 total isolates, respectively) and were detected from every subject, with a mean number of 8.6 [SD 2.0] organisms, the highest number being detected from groin swabs, 3.3 [SD 3.3] followed by nares 2.7 [SD 1.3] and oropharynx 2.6 [SD 1.3] (Table 1). PCR/ESI-TOF-MS identified 870 aerobic gram-positive bacteria (329 from groin swabs, 275 from nares, and 266 from oropharynx). The most common organisms (341) were CNS with numerous other gram-positive bacteria noted (Table 2). The *mecA* gene was identified in nearly every subject (96). No resistance elements of *vanA* and *vanB* were detected in this study.

**Table 2:** Number of healthy Soldiers colonized with aerobic gram-positive bacteria according to PCR/ESI-TOF-MS

Organism	Subjects^a^	Organisms by anatomic site (870 total)		
		Oropharynx	Nares	Groin
		266	275	329
*Abiotrophia defectiva*	2	2	0	0
*Abiotrophia elegans*	1	1	0	0
*Arthrobacter aurescens*	3	0	1	2
*Bacillus cereus*	2	0	0	2
*Bacillus clausii*	1	1	0	0
*Bacillus coagulans*	3	3	0	0
*Corynebacterium accolens*	1	0	1	0
*Corynebacterium auriscanis*	35	7	1	27
*Corynebacterium diphtheria*	1	1	0	0
*Corynebacterium falsenii*	16	0	1	15
*Corynebacterium jeikeium*	1	0	0	1
*Corynebacterium pseudodiphtheriticum*	38 (39)	1	35	3
*Corynebacterium sundsvallense*	1	0	0	1
*Corynebacterium urealyticum*	1	0	1	0
*Corynebacterium* spp.	11	0	3	8
*Enterococcus faecalis*	1	0	0	1
*Enterococcus faecium*	4	3	1	0
*Finegoldia magna*	18 (19)	5	7	7
*Gemella haemolysans*	1	1	0	0
*Gemella sanguinis*	4	3	1	0
*Granulicatella adiacens*	5	2	2	1
*Lactobacillus acidophilus*	1	1	0	0
*Lactobacillus casei*	3	3	0	0
*Lactobacillus crispatus*	1	0	0	1
*Lactobacillus delbrueckii*	5	1	2	2
*Lactobacillus gasseri*	1	0	0	1
*Lactobacillus helveticus*	4	3	1	0
*Lactobacillus johnsonii*	2	1	0	1
*Lactobacillus plantarum*	8	1	6	1
*Lactobacillus salivarius*	18 (19)	1	15	3
*Lactobacillus* sp.	1	1	0	0
*Lactobacillus vaccinostercus*	1	1	0	0
*Lysinibacillus sphaericus*	1	0	1	0
*Micrococcus luteus*	17 (19)	2	3	14
*Mycobacterium ulcerans*	1	0	1	0
*Nocardia asteroides*	45 (50)	1	30	19
*Nocardia farcinica*	1	0	0	1
*Staphylococcus aureus*	58 (80)	22	13	45
*Staphylococcus auricularis*	1	0	0	1
*Staphylococcus capitis/caprae*	7 (9)	4	3	2
*Staphylococcus epidermidis*	99 (201)	46	87	68
*Staphylococcus haemolyticus*	7	0	7	0
*Staphylococcus hominis*	80 (103)	23	7	73
*Staphylococcus lugdunensis*	4	1	2	1
*Staphylococcus saprophyticus*	9	2	4	3
*Staphylococcus warneri*	3 (4)	2	0	2
*Staphylococcus xylosus*	1	0	0	1
*Staphylococcus epidermidis/haemolyticus*	1	1	0	0
*Staphylococcus epidermidis/haemolyticus/hominis*	1	1	0	0
*Stomatococcus mucilaginosus*	26 (26)	21	2	3
*Streptococcus agalactiae*	27 (34)	6	14	14
*Streptococcus anginosus*	1	1	0	0
*Streptococcus constellatus*	1	0	1	0
*Streptococcus cristatus*	2	2	0	0
*Streptococcus gordonii*	8	4	4	0
*Streptococcus mutans*	3	3	0	0
*Streptococcus oralis*	5	5	0	0
*Streptococcus parasanguinis*	4	3	1	0
*Streptococcus peroris*	8	8	0	0
*Streptococcus pneumoniae*	54 (57)	46	11	0
*Streptococcus porcinus*	2	1	0	1
*Streptococcus pyogenes*	4	2	1	1
*Streptococcus sanguinis*	4	2	2	0
*Streptococcus suis*	8	5	1	2
*Streptococcus* spp.	9	6	2	1
*Streptococcus oralis/sanguinis*	2	2	0	0
*Streptococcus parasanguinis/pneumoniae*	1	1	0	0

^a^number of organisms listed in ( ) if not same as number of subjects

### Aerobic GNB

Aerobic GNB were also commonly found by PCR/ESI-TOF-MS in 80 subjects, with a mean number of 1.4 [SD 1.0] organisms, primarily the oropharynx followed by nares and groin (Table 1). In total, 140 aerobic GNB were identified (94 from oropharynx swabs, 32 from nares, and 14 from groin). The most common GNB identified were *Neisseria* spp. (59), followed by *Haemophilus influenzae* (21), *Moraxella* spp. (19), and *Escherichia coli* (7) (Table 3). As was seen with gram-positive bacteria, several GNB known to cause human disease were frequently found (Table 3). No genes commonly associated with GNB resistance were detected by PCR/ESI-TOF-MS.

**Table 3:** Number of healthy Soldiers colonized with aerobic gram-negative bacteria according to PCR/ESI-TOF-MS

Organism	Subjects^a^	Organisms by anatomic site (140 total)		
		Orophaynx	Nares	Groin
		94	32	14
*Acinetobacter* spp.	3	2	0	1
*Actinobacillus pleuropneumoniae*	1	1	0	0
*Akkermansia muciniphila*	1	0	1	0
*Bordetella petrii*	1	0	0	1
*Brevundimonas diminuta*	1	1	0	0
*Burkholderia cenocepacia*	2	0	1	1
*Buttiauxella* spp.	2	2	0	0
*Campylobacter concisus*	1	1	0	0
*Campylobacter fetus*	1	1	0	0
*Campylobacter hyointestinalis*	1	1	0	0
*Campylobacter mucosalis*	2	2	0	0
*Eikenella corrodens*	1	0	1	0
*Enterobacter aerogenes*	1	0	0	1
*Escherichia coli*	7	4	3	0
*Haemophilus influenzae*	20 (21)	18	3	0
*Haemophilus* sp.	1	1	0	0
*Klebsiella pneumoniae*	4	0	2	2
*Moraxella atlantae*	2	0	0	2
*Moraxella catarrhalis*	9 (10)	1	9	0
*Moraxella catarrhalis/nonliquefaciens*	4 (5)	1	4	0
*Moraxella lacunata*	1	0	1	0
*Moraxella* sp.	1	1	0	0
*Mycoplasma hominis*	1	0	0	1
*Neisseria canis*	19 (20)	17	2	1
*Neisseria flava*	13	9	2	2
*Neisseria flavescens*	3	3	0	0
*Neisseria gonorrhoeae*	2	1	0	1
*Neisseria meningitidis*	2	2	0	0
*Neisseria mucosa*	3	3	0	0
*Neisseria sicca*	1	1	0	0
*Neisseria weaveri*	1	0	0	1
*Neisseria* spp*.*	4	4	0	0
*Pasteurella multocida*	6	5	1	0
*Porphyromonas endodontalis*	1	1	0	0
*Proteus mirabilis*	3	1	2	0
*Raoultella planticola*	2	2	0	0
*Rickettsia* sp.	1	1	0	0
*Salmonella agona*	1	1	0	0
*Salmonella enterica*	1	1	0	0
*Salmonella matopeni*	2	2	0	0
*Salmonella* spp.	2	2	0	0
*Tatumella ptyseos*	1	1	0	0

^a^number of organisms listed in ( ) if not same as number of subjects

### Anaerobic bacteria

Multiple anaerobic bacteria were detected by PCR/ESI-TOF-MS in 75 subjects, with a mean number of 1.2 [SD 0.98] organisms; the most common site being the nares (56 subjects – mean number of 0.6 [SD 0.6] organisms) (Table 1). In total, 96 anaerobic gram-positive bacteria (6 from oropharynx swabs, 57 from nares, and 33 from groin) and 27 anaerobic GNB were identified (23 from oropharynx swabs, 3 from nares, and 1 from groin). *Propionibacterium acnes* was the most commonly identified anaerobic gram-positive bacteria (70) (Table 4).

**Table 4:** Number of healthy Soldiers colonized with anaerobic bacteria according to PCR/ESI-TOF-MS

Organism	Subjects^a^	Organisms by anatomic site		
		Oropharynx	Nares	Groin
Total gram-negative organisms (27)		23	3	1
* Bacteroides capillosus*	7 (8)	7	1	0
* Bacteroides fragilis*	3 (4)	3	0	1
* Bacteroides thetaiotaomicron*	5	4	1	0
* Fusobacterium necrophorum*	2	2	0	0
* Fusobacterium nucleatum*	6	6	0	0
* Treponema denticola*	2	1	1	0
Total gram-positive organisms (96)		6	57	33
* Propionibacterium acnes*	59 (70)	2	49	19
* Propionibacterium granulosum*	2	1	0	1
* Bifidobacterium inopinatum*	12	0	8	4
* Bifidobacterium longum*	1	0	0	1
* Bifidobacterium subtile*	7	0	0	7
* Bifidobacterium thermophilum*	1	0	0	1
* Clostridium beijerinckii*	2	2	0	0
* Clostridium novyi*	1	1	0	0

^a^number of organisms listed in ( ) if different from number of subjects

PCR/ESI-TOF-MS identified fungal species in 46 subjects with a mean of 0.60 [SD 0.79] organisms per individual (Table 1). A total of 61 fungal organisms, with 39 in the oropharynx, 14 nares, and 8 groin were detected (Table 5).

**Table 5:** Number of healthy Soldiers colonized with fungi according to PCR/ESI-TOF-MS

Organism	Subjects^a^	Organisms by Anatomic Site (61 total)		
		Oropharynx	Nares	Groin
		39	14	8
*Alternaria alternate*	11 (15)	2	10	3
*Aureobasidium pullulans*	3	1	1	1
*Candida albicans*	6	5	0	1
*Candida parapsilosis*	3 (5)	3	1	1
*Candida tropicalis*	1	1	0	0
*Saccharomyces paradoxus*	1	1	0	0
*Saccharomyces cerevisiae/paradoxus*	29 (30)	26	2	2

^a^number of organisms listed in ( ) if not same as number of subjects

### PCR/ESI-TOF-MS compared with traditional culture for gram-negative bacteria

Traditional culture identified colonization of 34 sites from 23 subjects with GNB. The most commonly recovered organism was *Pseudomonas aeruginosa* with 4 isolates, all of which were from oropharyngeal sites. Other GNB included *E.coli* (3 isolates), *K. pneumoniae* (3 isolates), and *Serratia marcescens* (3 isolates). A complete list of less common GNB is shown in Table 6. Traditional culture also did not detect any MDR GNB.

**Table 6:** Comparison of gram-negative bacterial colonization by traditional culture and PCR/ESI-TOF-MS per subject

Organism	Nares		Oropharynx		Groin	
	Culture	ESI-MS	Culture	ESI-MS	Culture	ESI-MS
Total organisms	4	7	17	10	13	4
*Acinetobacter baumannii-calcoaceticus* complex	0	0	1	0	0	0
*Acinetobacter lwoffii*	0	0	1	0	1	0
*Acinetobacter species*	0	0	0	2	0	1
*Alcaligenes* sp.	0	0	1	0	0	0
*Citrobacter* sp.	0	0	0	0	0	0
*Enterobacter aerogenes*	1	0	0	0	1	1
*Enterobacter cloacae*	0	0	1	0	0	0
*Escherichia coli*	2	3	0	4	1	0
*Klebsiella oxytoca*	0	0	1	0	1	0
*Klebsiella pneumoniae*	0	2	1	0	2	2
*Moraxella* sp.	0	0	0	1	0	0
*Ochrobactrum anthropi*	0	0	2	0	0	0
*Pantonea agglomerans*	0	0	0	0	2	0
*Proteus mirabilis*	0	2	0	1	0	0
*Pseudomonas aeruginosa*	0	0	4	0	0	0
*Pseudomonas fluorescens*	0	0	1	0	0	0
*Pseudomonas oryzihabitans*	0	0	0	0	1	0
*Pseudomonas* species	0	0	1	0	0	0
*Pseudomonas stutzeri*	0	0	0	0	1	0
*Salmonella* spp.	0	0	0	2	0	0
*Shigella flexneri*	0	0	0	0	1	0
*Serratia marcescens*	1	0	1	0	1	0
*Shewanella putrefaciens*	0	0	0	0	1	0
*Stenotrophomonas maltophilia*	0	0	2	0	0	0

PCR/ESI-TOF-MS found more GNB isolates than traditional culture (80 subjects versus 23 subjects). When comparing the organisms found by PCR/ESI-TOF-MS and traditional culture, only one non-MDR *E. coli* was identified by both systems from the same subject and collection site (nares). Similarly, neither method detected any MDR GNB. Spearman correlation between GNB on traditional culture versus PCR/ESI-TOF-MS for nares was 0.68, oropharynx was 0.44, and groin was 0.13.

## Discussion

Pathogen detection has several limitations within the current practices of traditional microbiological methods. Newer molecular diagnostic methods, such as PCR/ESI-TOF-MS, offer potential advantages over traditional microbiological methods in detecting microbial pathogens. One potential limitation of traditional microbiology is the inability to culture specific organisms in the laboratory due to difficulty in replicating the perfect media for growth while molecular platforms, such as PCR/ESI-TOF-MS, circumvent this by focusing on the genetic elements present in a sample for organism identification. In this study, PCR/ESI-TOF-MS detected many clinically relevant bacteria (e.g., *Neisseria meningitidis, N. gonorrhoeae, N. asteroides, S. pneumoniae, Fusobacterium necrophorum*) that are typically associated with virulent infections in a population without evidence of clinical disease, and identified multiple isolates of more fastidious organisms such as *N. asteroides* and several nutritionally deficient *Streptococcus* spp.

PCR/ESI-TOF-MS detected large numbers of isolates of expected commensal organisms, especially among the aerobic gram-positive bacteria, with CNS being most commonly identified. The carriage rate of *S. pneumoniae* using standard microbiological methods has previously been reported in adult populations, varying in reported prevalence from 3.7 to 38 % [16, 17]. Among a healthy, primarily young adult population in this study, the *S. pneumoniae* carriage rate was found to be 53.5 %. This is significantly higher than previously reported and is similar to carriage rates previously shown in young children populations (53–67 %) [16, 17] *S. aureus* was also commonly found in 57.4 % of individuals sampled in this study, consistent with previously reported colonization rates between 32.7 and 53 % [18–23] *Streptococcus agalactiae* was found on 26.7 % of subjects in this study. While *S. agalactiae* has been evaluated previously in vaginal and anorectal colonization studies; there have been limited colonization studies looking at carriage rates in other anatomic sites. The reported colonization rate has varied from 8.5 to 32.9 % depending on the study population and geographic location [24–28].

In addition to colonization with expected organisms, PCR/ESI-TOF-MS also detected traditionally pathologic aerobic gram-positive bacteria such as *N. asteroides* in a high percentage of the study population in absence of clinical disease. *N. asteroides,* which traditionally has not been thought of as part of the body’s normal flora [29], was detected in 44.5 % of the study population, most commonly from the nares (30 isolates) and groin (19 isolates). While this is surprisingly high, *Nocardia* has been difficult to grow and identify by traditional culture historically and, for this reason, other direct molecular detection methods have been evaluated for enhanced identification of *Nocardia* [30, 31]. Case series have previously shown *Nocardia* colonization of the respiratory tract without clinical or radiographic findings consistent with disease, however all these patients had underlying lung disease [32, 33].

PCR/ESI-TOF-MS also found a small number of *Streptococcus* species, formerly known as nutritionally deficient Streptococci*,* such as *Granulicatella adiacens*, *G. sanguinis, Abiotrophia defectiva, Gemella haemolysans*, *A. elegans* which, similar to *N. asteroides,* are difficult to isolate but associated with disease including endocarditis. Although infrequently studied, *Abiotrophia, Granulicatella,* and *Gemella* have been shown, using molecular methods, to account for a significant part of the human oral microbiome [7, 34].

Genetic resistance elements common to gram-positive bacteria were also examined, and the *mecA* gene was found in nearly every subject (96/101 subjects). *Staphylococcus* resistance was felt to be a broader study question comparing PCR/ESI-TOF-MS with detection by traditional culture, and has been previously published; therefore, the data are not presented in this manuscript [14]. Other specific genetic markers of resistance, such as the *vanA* and *vanB* genes, were not identified in our study isolates. The significance of these resistance markers in terms of clinical diagnosis and therapy is unclear. Clinical decision making regarding decolonization might be affected by the presence of genetic resistance elements, particularly if they are transferred between pathogens co-located on the body.

Aerobic GNB findings were similar to those of gram-positive bacteria, such as PCR/ESI-TOF-MS detected large numbers of expected colonizing organisms (e.g., *H. influenzae* and *Moraxella catarrhalis*), with fewer numbers of organisms associated with clinical virulence in healthy individuals. In this study of healthy military personnel, the *H. influenzae* carriage rate was determined to be 19.8 % and the *M. catarrhalis* carriage rate was 8.9 %. In a previous carriage study of *H. influenzae* and *M. catarrhalis* in healthy, asymptomatic individuals*,* adult carriage rates were 23 and 17 %, respectively [15]. PCR/ESI-TOF-MS also identified important GNB human pathogens such as *Burkholderia cenocepacia*, *N. gonorrhoeae, N. meningitides,* and *Rickettsia sp.* which again are not thought of as traditional colonizing organisms. No genetic resistance elements for GNB were found in this study.

While PCR/ESI-TOF-MS did identify some unexpected GNB, it identified only a few *Acinetobacter* spp. (3 subjects) and no *P. aeruginosa,* which are two examples of potential MDR organisms. There are limited numbers of studies using similar populations, however a study looking at surveillance of organisms in a burn intensive care unit and orthopedic ward found a higher prevalence of *Acinetobacter* and *P. aeruginosa* [13]. While outpatient colonization rates of *A. baumannii-calcoaceticus* (ABC) have been variable, U.S. Army soldiers without significant healthcare exposure have been shown to have lower carriage rates, whereas hospitalized soldiers have much higher rates of colonization [15, 35–38].

Similar to aerobic bacteria, anaerobic bacteria and fungi known to be members of the colonizing human microbiota were found with PCR/ESI-TOF-MS. *P. acnes* was the most commonly detected anaerobic gram-positive bacteria, *Bacteroides* spp. the most common anaerobic GNB, and *Saccharomyces cerevisiae/paradoxus* the most common fungus. Notably, *Fusobacterium* spp.*,* commonly thought to be pathogenic organisms, were isolated in 8 asymptomatic, healthy subjects.

A limitation of PCR/ESI-TOF-MS in this study, that may warrant further evaluation prior to broader application, was the limited anatomic site-correlation between screening methods. Only one pathogen was identified by both systems in the same subject from the same anatomic site (*E.coli* from the nares). This discordance has been observed elsewhere, with one study reporting a relatively high false negative rate with PCR/ESI-MS when trying to resolve mixtures [39]. There are several potential reasons for this discordance. With molecular platforms, such as PCR/ESI-TOF-MS, there is a greater uncertainty as to what is being sampled. Some investigators have suggested a role for preferential amplification, where the DNA primers are saturated from the most abundant organisms present, causing organisms in smaller quantity to be missed [40]. Another limitation is that we did not screen for all gram-positive bacteria, fungi, or anaerobes with traditional culture for comparison. In addition, this pilot study also evaluated only a limited number of anatomic sites per individual, which may have led to an underestimate of the total number and diversity of potential isolates from our study participants – in fact microbiome studies have identified a high degree of heterogeneity among nearly all anatomic sites. The lack of additional confirmatory tests (e.g., a second molecular diagnostic platform) to validate the PCR/ESI-TOF-MS findings is also a potential study limitation. Furthermore, a limited number of subjects were examined in one particular environment and study participants were only screened at a single time point, limiting our ability to determine if individuals were only transiently colonized. Future studies evaluating individuals over a period of time would be helpful in determining the incidence and prevalence of true colonization. Validation of this molecular method as a colonization screening tool for a broad array of pathogens might be particularly useful for patients at higher risk for infection, such as immunosuppressed or burn patients.

## Conclusions

In summary, the PCR/ESI-TOF-MS detected large numbers of isolates of expected commensal organisms in a population of healthy, asymptomatic individuals, but it also detected many bacteria (e.g., *N. meningitidis, N. gonorrhoeae, N. asteroides, S. pneumoniae, Fusobacterium* spp.) that are often associated with clinically virulent infections suggesting that presence alone does not trigger virulence [1]. This molecular platform identified higher carriage percentages for pathogenic bacteria than have been previously shown in the literature, and identified multiple organisms that have traditionally been difficult to culture in the laboratory. Additional advantages of this molecular platform are the potential for increased sample throughput with decreased processing times. Further validation with confirmatory molecular technologies should be performed to ascertain the clinical significance of identified organisms and appropriate clinical application.

## Abbreviations

GNB, gram negative bacteria; MRSA, methicillin-resistant Staphylococcus aureus; MSSA, methicillin-susceptible Staphylococcus aureus; PCR/ESI-TOF-MS, polymerase chain reaction/electrospray ionization-time-of-flight-mass spectrometry
